# Smartphones through children’s eyes: perceived benefits and educational considerations

**DOI:** 10.3389/fpsyg.2025.1596595

**Published:** 2025-06-19

**Authors:** Mirari Gaztañaga, Nahia Idoiaga-Mondragon, Idoia Legorburu Fernandez, Amaia Eiguren Munitis

**Affiliations:** ^1^Department of Basic Psychological Processes and Their Development, University of the Basque Country UPV/EHU, Donostia-San Sebastian, Spain; ^2^Department of Evolutionary and Educational Psychology, University of the Basque Country UPV/EHU, Leioa, Spain; ^3^Department of Didactics and School Organization, University of the Basque Country UPV/EHU, Leioa, Spain

**Keywords:** child-centered research, smartphone use, digital education, children’s perspectives, lexicometric analysis, well-being, boredom, mobile technology

## Abstract

**Introduction:**

Smartphones have become central to everyday life, especially among children and adolescents. While they provide opportunities for communication, learning, and entertainment, growing concerns persist regarding their association with negative outcomes such as anxiety, depression, and reduced social and cognitive development. Despite the prevalence of these concerns, children’s own perspectives are often overlooked in debates about smartphone regulation and education.

**Methods:**

This mixed-methods study involved a total of 642 children from the Basque Country. Data collection combined quantitative and qualitative approaches to explore children’s perceptions of smartphone use. Participants shared their experiences and opinions through structured questionnaires and open-ended responses, allowing for both statistical analysis and thematic exploration.

**Results:**

Children identified four main benefits of smartphone use: (1) maintaining social connections and contacting parents in emergencies, (2) accessing information, (3) reducing boredom, and (4) engaging in multimedia entertainment. Ownership status influenced perceived benefits: those with personal devices highlighted communication and academic uses, while those using parents’ devices focused on entertainment. The study also examined the role of smartphone education in schools and family discussions, revealing significant correlations with children’s reported benefits.

**Discussion:**

Findings underscore the importance of incorporating children’s voices into digital policy and educational strategies. The study highlights the need for early digital education programs that promote balanced and responsible use. Additionally, it identifies boredom as a potentially constructive driver for creative and emotional development. Adopting a child-centered framework for understanding smartphone use can foster more effective and empathetic approaches to digital wellbeing.

## 1 Introduction

Smartphones have become an essential aspect of modern society, gradually increasing their presence to the extent that they have become a dominant presence in our personal and social lives (Oksman and Rautiainen, 2017). This trend is particularly evident among children and adolescents (Armakolas et al., 2024). Research has indicated that most adolescents acquire their first smartphone at around the age of 12 (Girela-Serrano et al., 2022; Richter et al., 2022), though smartphone use often begins years before ownership. A 2013 study revealed that 72% of children aged 0 to 8 had used a smartphone for activities such as playing games, watching videos, communicating, taking pictures, or accessing applications (Rideout et al., 2013). Furthermore, 38% of children aged 0 to 2 had experienced using a smartphone (Rideout et al., 2013). Irrespective of ownership, there has been a substantial rise in the use of smartphones among children and young people in recent years (Kopecký et al., 2021).

This issue remains highly controversial, as recent research suggests that smartphone use can have adverse psychological, physical, and social effects despite ongoing technological advancements (Girela-Serrano et al., 2022). In response, global organizations such as the World Health Organization (WHO) have established clear guidelines on screen time for young children. The WHO strongly recommends that children under 2 avoid screen time entirely, while those aged 2 to 5 should be limited to a maximum of 1 h per day (World Health Organization, 2019). More recently, in December 2024, the Spanish Society of Paediatrics recommended that children under the age of 6 should not be exposed to screens, citing adverse effects in areas such as sleep, cardiovascular risk, brain volume, and nutrition (Asociación Española de Psiquiatría, 2024).

The appropriate age for smartphone ownership remains unclear; however, some experts suggest that 13–14 is a suitable milestone (Bouchrika, 2025; Richter et al., 2022). By this age, children’s prefrontal cortex has developed sufficiently to allow for improved decision-making, enhanced reasoning abilities, and better impulse control and inhibition (Casey et al., 2011). However, it is crucial to consider individual maturity, responsibility, and understanding of limitations beyond age alone.

To comprehend the increasing prevalence of smartphone use among adults and children, it is essential to recognize the numerous advantages that these devices offer (Sharma et al., 2022; Li and Chan, 2022). Initially, mobile phones were used solely for voice communication and text messaging. However, they have since evolved to include cameras, calculators, voice recorders, gaming devices, and music players. Today, smartphones serve as gateways to the virtual world, providing internet access and facilitating various information, communication, and entertainment opportunities (Soukup, 2015). The advent of social networking platforms such as Facebook, TikTok, WhatsApp, and X has enabled global connectivity, online shopping, and the utilization of various applications that have the potential to simplify and enhance daily life (Wieland, 2014; Sahu et al., 2019). The benefits of these technologies include instant communication, access to entertainment and information at any time and in any place, time management, and the maintenance of social identity (Armakolas et al., 2024; Bian and Leung, 2015; Kuss et al., 2018; Kwon et al., 2013; Lin et al., 2014).

For children and adolescents, smartphone advantages mirror those observed in adults. During adolescence, social connectivity is a frequently cited benefit (O’reilly, 2020; Peng et al., 2025; Schnauber-Stockmann et al., 2021). As children grow, their social circles expand, and they use smartphones to communicate with friends via calls, texts, and social networking sites, fostering feelings of social connectedness (Dharejo et al., 2023).

Smartphones also serve as a source of recreational activity, offering access to multimedia content and games. Children can consume digital content passively or create their own, an increasingly common practice that nurtures their creativity (Aznar et al., 2019; Kopecký et al., 2020, 2021). Additionally, smartphones provide educational opportunities through interactive learning apps covering subjects such as languages, math, and history (Metruk, 2022; Straková and Cimermanová, 2018; Woodcock et al., 2012). Some schools have integrated digital resources into their curricula, recognizing their value in enhancing learning experiences. For instance, research indicates that health apps can help children manage obesity by improving diet and physical activity, ultimately reducing body mass index (Vaidya et al., 2025).

From a parental perspective, smartphones offer tracking and monitoring capabilities, providing children with a degree of autonomy while ensuring parental reassurance regarding their whereabouts (Davis et al., 2024). Additionally, smartphones facilitate communication between children and family members, enhancing familial dialogue and cohesion (Devitt and Roker, 2009).

Despite these benefits, concerns remain about whether children and young people should rely solely on smartphones for entertainment (Li et al., 2018; Liu and Lu, 2022). While smartphones facilitate communication with friends and family, it is important to assess whether they should be the primary or exclusive medium for social interaction. Numerous studies have highlighted the risks associated with smartphone use (Girela-Serrano et al., 2022; Syvertsen et al., 2022). Excessive screen time has been shown to alter brain development in children, leading to reduced cognitive abilities (National Institute of Health, 2018). Additionally, prioritizing digital interaction over face-to-face communication has been linked to impaired social skills in children (Valdesolo, 2015). Research also suggests a correlation between social media use and mental health issues such as depression and anxiety (Girela-Serrano et al., 2022), contributing to increased cases of internet addiction and related disorders (Moreno-Guerrero et al., 2020; Saquib, 2020; Tateno et al., 2019). Furthermore, evidence indicates that social media can negatively impact young people’s self-esteem and self-concept (Rodríguez et al., 2019). A particularly concerning risk is cyberbullying, which has become increasingly prevalent in recent years (Kopecký and Szotkowski, 2017; Álvarez-García et al., 2017).

Despite the well-documented negative effects of smartphone use on children, their adoption of these devices continues to rise (Kopecký et al., 2021). To effectively regulate, support, educate, and explore alternative smartphone uses, it is essential to first understand their appeal to children and the perceived benefits they derive from them. To our knowledge, no research has openly gathered children’s perspectives on the advantages of smartphone use, allowing them to express themselves freely. Without considering their viewpoints, it is impossible to develop meaningful educational responses to the issue of children’s smartphone use. In other words, including the voices of those directly affected is crucial. Therefore, this study aims to explore children’s perceptions, identifying the benefits they associate with smartphone usage.

## 2 Methodology

This study employed a rigorous and systematic approach to collecting both qualitative and quantitative data. The primary data collection method was online questionnaires specifically designed for this research. These surveys incorporate a mix of open-ended and closed-ended questions, ensuring a comprehensive exploration of participants’ perspectives. This combination allows for both measurable data and contextual insights, providing a well-rounded analysis of the target demographic.

### 2.1 Sample

This study included 634 children from a region of the autonomous community of the Basque Country (northern Spain). Participants had a mean age of 9.33 years (SD = 1.97) (age range 6–16). Regarding gender distribution, 52.9% identified as female, 46.8% as male, and 0.3% as non-binary. The majority of participants were in fifth grade (10–11 years, 20.4%, *n* = 129), followed by sixth grade (11–12 years, 18.3%, *n* = 116), fourth grade (9–10 years, 15.9%, *n* = 101), third grade (8–9 years, 15.8%, *n* = 100), second grade (7–8 years, 12.3%, *n* = 78), first grade (6–7 years, 11.2%, *n* = 71) and compulsory secondary education (12–16 years, 6.2%, *n* = 39). Regarding smartphone use, 27.3% (*n* = 173) of children owned a smartphone, 65.5% (*n* = 415) used their parents’ smartphone, and 6.8% (*n* = 45) did not use one.

### 2.2 Procedure

Before data collection, approval was obtained from the ethics committee of the University of the Basque Country [M10_2024_073]. After receiving detailed information about the research procedures, all participants and their parents/guardians provided written informed consent. Participants were recruited using a non-probabilistic snowball sampling method. Researchers created an online questionnaire, which was distributed through schools. Children completed the questionnaire themselves, while parents transcribed their responses for those unable to write.

### 2.3 Instrument

The questionnaire consisted of two distinct sections. The first section collected key socio-demographic data, including participants’ age, gender (“Girl,” “Boy,” or “Non-binary”), school year, and smartphone usage (owning a smartphone, using an adult’s smartphone, or not using one). It also included questions about exposure to smartphone training in schools (“Yes, more than once,” “Yes, once,” or “No, never”) and discussions with parents about smartphone use (“Numerous occasions,” “Occasionally,” “Very rarely,” or “Never”). The second section explored children’s perceptions of smartphone use. Participants were asked to identify and describe four benefits or “good things” about smartphones, providing explanations where relevant. These responses served as the foundation for further analysis, offering valuable insights into children’s perspectives.

### 2.4 Analysis

Open-ended responses are best analyzed using the Reinert method (Reinert, 1983), facilitated by Iramuteq software. This method is widely recognized for its effectiveness in analyzing children’s open-ended responses across various disciplines (Legorburu et al., 2022; Idoiaga Mondragon et al., 2021) and has been shown to successfully address reliability and validity issues in text analysis (Klein and Licata, 2003). The Reinert method employs a top-down hierarchical cluster analysis, extracting thematic classes based on statistical indicators such as typical words and text segments (Idoiaga and Belasko, 2019). Iramuteq identifies words and text segments with the highest chi-square values, highlighting those that best represent each class or frequently mentioned idea by participants.

Following previous applications of the Reinert method (Camargo and Bousfield, 2009), the raw data were entered into Iramuteq. Significant vocabulary items within each class were selected based on three criteria: (1) an expected word value exceeding 3; (2) chi-square statistical evidence of association with the class (*χ*^2^ ≥ 3.89, *p* = 0.05, df = 1); and (3) the word predominantly appearing in that class with a frequency of 50% or more. The software also identified text segments associated with each class, ranking them according to their chi-square values. Text segments with the most significant chi-square values in each class were then selected.

Once these “lexical universes” were identified, they were linked to “passive” (independent) variables, including gender, school year, type of smartphone use, prior training on mobile phone use, and discussions with parents about mobile phone use. The analyst then derived thematic classes consisting of typical words and text segments (quotes) with the highest chi-square values. These classes served as the foundation for interpreting the lexical worlds.

The Reinert method produces statistical, transparent, and reproducible data up to the point of interpretation, where the analyst assigns labels. In the final phase, two researchers independently named each class based on associated words and text segments. A third researcher then finalized the labels, which were approved by all three researchers.

Quantitative data were analyzed using IBM SPSS version 28 (Armonk, NY, USA).

## 3 Results

### 3.1 Children’s Main perceived benefits of smartphone use

**Figure 1:**
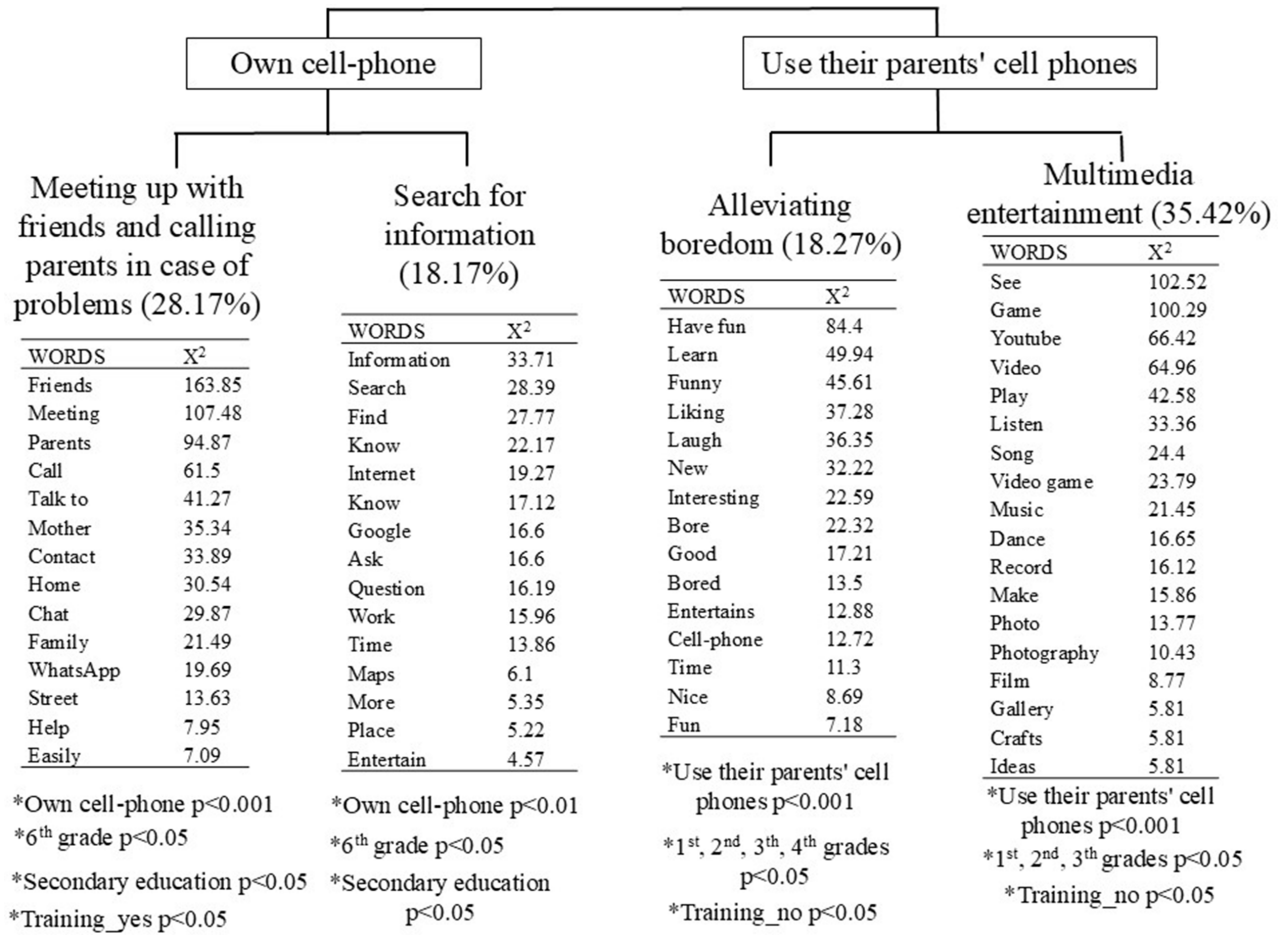
The hierarchical clustering dendrogram showing the most frequent words and those with the greatest association *χ*^2^ (1), *p* < 0.001 extracted by the Reinert method.

Using descending hierarchical analysis, the Reinert method was applied to identify children’s perceptions about the benefits of using smartphones. Each issue or concept is represented by a set of characteristic words and text segments, forming a “class.” The analysis segmented the corpus into 634 sections, resulting in four distinct classes, as illustrated in Figure 1. These classes will be examined individually in the following sections.

As demonstrated in Figure 1, the analysis yielded four distinct classes of responses. Two of these classes pertain to the advantages of smartphones for children, as reported by children who possess their own smartphones: “Socializing with friends and calling parents in case of problems” and “Searching for information.” The remaining two classes relate to the use of smartphones by children who share their parents’ devices, including “Alleviating boredom” and “Multimedia entertainment.”

The largest class (28.17%) highlighted that smartphones facilitate social interaction and communication with parents in emergencies. Children reported using WhatsApp to chat with friends and emphasized the importance of contacting parents when needed. A statistically significant relationship was identified between this perception and smartphone ownership (*p* < 0.001), as well as being in 6th grade or secondary school (*p* < 0.05) and receiving smartphone training in school (*p* < 0.05).

Sample responses include:

- *The best thing is that I can meet my friends by and chat with them by WhatsApp and I can also call my parents if I need anything (Girl, 6th grade, χ^2^ = 491.46).*
- *Especially to talk to or chat to my friends, at home or in the street. And if I’m with my friends and I want to go home, I will not be waiting like before — I call my parents and they come and pick me up (Girl, Secondary education, χ^2^ = 452.16).*
- *I can call my mother, meet with my friends, play games, and easily call my parents for help (Boy, 6th grade, χ^2^ = 446.95).*
- *I find it good for meeting or chatting with friends and going out. It is very important to have it to be able to communicate or to call my parents when I have problems (Girl, Secondary education, χ^2^ = 416.63).*
- *For chatting with friends, we mostly talk on our mobile phones, even when we are on the street, so that we can talk to other groups, etc. (Girl, Secondary education, χ^2^ = 389.75).*

The second benefit highlighted by the children, accounting for 18.17% of the total, relates to the ease of retrieving information using smartphones. Children emphasized how they can quickly search for information using Google, particularly for academic purposes and locating places via Google Maps. Additionally, they acknowledged that smartphones provide access to information they might not feel comfortable requesting from others or that is not readily available through other means. This trend was more common among children who owned a smartphone (*p* < 0.01), those in sixth grade (*p* < 0.05), and those in secondary school (*p* < 0.05).

Sample responses include:

- *You can Google, for example, the meaning of a word or information about whatever you want and find out the results of football matches (Boy, 6th grade, χ^2^ = 315.15).*
- *There are some things we do not know, and Google has information on all topics. We can search for anything or anywhere (Girl, 6th grade, χ^2^ = 303.31).*
- *With Google Maps, you can search for sites and with Google information, so you do not have to ask anyone, for work or for yourself, to find out anything (Boy, 6th grade, χ^2^ = 299.28).*
- *You can search for information to do work or about things that interest you and you do not want to ask others. You can learn a lot of things from the videos (Boy, 6th grade, χ^2^ = 287.30).*

The third benefit of smartphones, accounting for 18.27% of the total, pertains to using smartphones as a tool for alleviating boredom. Children described smartphones as highly engaging and entertaining devices that effectively pass the time. This trend was more commonly reported among children who used their parents’ smartphones (*p* < 0.001), particularly those in 1st through 4th grade (*p* < 0.05), and those who had not received formal training on smartphone use in school (*p* < 0.05).

The most significant responses include:

- *I learn new and interesting things, have fun, and do not get bored (Girl, 3rd grade, χ^2^ = 235.98).*
- *If I get bored, I learn new dances and nice and funny handicrafts. I like ninja kids (Girl, 3rd grade, χ^2^ = 208.20).*
- *Time goes by fast, playing. I’ve learned a lot; I always have fun, and I do not get bored (Boy, 4th grade, χ^2^ = 154.96).*
- *That time goes by very fast, and parents get angry (Boy, 4th grade, χ^2^ = 67.57)*
- *You do not get bored playing the games! It’s fun because you can do lots of new things, and time flies by! (Boy, 1st grade, χ^2^ = 147.54).*

The final class, accounting for 35.42% of the total responses, highlights the role of smartphones in multimedia entertainment. Children noted that smartphones serve as versatile tools for watching YouTube videos, playing video games, listening to music, taking and editing photos, recording videos, and exploring crafts. This benefit was predominantly mentioned by children using their parents’ devices (*p* < 0.001) across various elementary grade levels (*p* < 0.05), including early childhood education (*p* < 0.05), and those without formal smartphone training in school (*p* < 0.05).

The most prevalent phrases employed by the children to articulate this concept were:

- *I like to watch videos I like on YouTube, play a lot of games, dance, and listen to music on my mobile phone (Girl, 2nd grade, χ^2^ = 278.25).*
- *I can laugh watching videos, play video games, listen to music in the car, and even watch a film (Boy, Secondary education, χ^2^ = 268.52).*
- *I like to listen to music, watch video clips on YouTube, call, send messages, and learn how to make handicrafts (Girl, 1st grade, χ^2^ = 258.47).*
- *I can watch YouTube, play games, use Snapchat, and take photos and videos! (Boy, 2nd grade, χ^2^ = 173.66).*

### 3.2 Prior training received by children on the use of smartphones

Regarding smartphone use training in schools, 38% of children (*n* = 244) reported receiving instruction on multiple occasions, 15.6% (*n* = 100) had a single instructional session, and 46.5% (*n* = 298) stated they had never received any formal instruction. A significant relationship was found between a child’s school level and the amount of training received (*X*^2^ = 149.40 (14), *p* < 0.001), with the critical point observed in the fourth year of elementary school, as younger children had received minimal training (see Table 1).

**Table 1:** Training received according to school year.

	ECE	1 grade	2 grade	3 grade	4 grade	5 grade	6 grade	CSE	Total
Yes, more than once	0	8	15	21	47	63	70	20	**244**
Yes, once	1	8	5	7	19	32	19	9	**100**
No, never	8	54	58	72	35	34	27	10	**298**
*Total*	***9***	***70***	***78***	***100***	***101***	***129***	***114***	***39***	***642***

ECE, Early childhood education (2–6 years); CSE, Compulsory secondary education (12–16 years). The italic value only indicates the title of the total number of children according to each school year. The bold values are the total values of children in each school year; and the total numer of responses for numerous occasions, occasional basis, very rarely, and never.

Additionally, discussions about smartphone use with parents varied widely. Among the children surveyed, 42.2% (*n* = 271) reported frequent discussions with their parents, 36.3% (*n* = 233) had occasional conversations, 15.7% (*n* = 101) rarely discussed the topic, and 5.8% (*n* = 37) never engaged in such discussions. A significant relationship was found between these discussions and the child’s school grade (*X*^2^ = 55.56 (12), *p* < 0.001) (see Table 2).

**Table 2:** Discussion of smartphone usage with parents according to school year.

	ECE	1 grade	2 grade	3 grade	4 grade	5 grade	6 grade	CSE	Total
Numerous occasions	0	21	34	47	33	51	63	22	**271**
Occasional basis	4	25	23	32	39	53	42	15	**233**
Very rarely	3	17	11	14	22	2	10	2	**101**
Never	2	7	10	7	7	3	1	0	**37**
*Total*	***9***	***70***	***78***	***100***	***101***	***129***	***116***	***39***	***642***

ECE, Early childhood education (2–6 years); CSE, Compulsory secondary education (12–16 years). The italic value only indicates the title of the total number of children according to each school year. The bold values are the total values of children in each school year; and the total number of responses for numerous occasions, occasional basis, very rarely, and never.

## 4 Discussion

The objective of this research was to understand the benefits of smartphone use from the perspective of the children themselves, with the aim of facilitating a smoother transition to digitalization (Nansen, 2020). The findings provide valuable insights, allowing for a deeper exploration of the issue.

A key observation in this study is that the children’s responses fall into two distinct categories: responses from those who own a smartphone (predominantly older children) and those who do not (typically younger children). Among the children who own a smartphone, two primary benefits emerge: communication and access to information. Smartphones have become essential tools for older children to socialize with friends, whether through conversations, messaging, or other forms of digital interaction (Johnsen, 2017; Thulin and Vilhelmson, 2017). However, this dynamic can create social disparities, as children without smartphones may feel excluded from peer interactions, leading to potential social alienation (Davie et al., 2004). This is particularly concerning during the early stages of adolescence when peer groups assume a pivotal role in the lives of these individuals (Kindschi et al., 2019). In the context of digital socialization, this phenomenon warrants careful examination. During adolescence (ages 11 to 16), young people undergo a critical transition to adulthood, marked by significant social development (Hurrelmann and Bauer, 2018). At this stage, children need to build social relationships independently, without constant adult supervision. They should also be able to communicate effectively with peers and interpret both verbal and non-verbal cues essential to the communication process (Mantilla, 2007). However, when peer communication shifts from direct, face-to-face interactions to digital exchanges—such as chatting and written messages that follow new communication codes (Gómez-Camacho et al., 2023)—the socialization process may be affected (Smith et al., 2015).

Online communication is often prone to misinterpretation, a particularly pronounced challenge among adolescents (Shubhra and Krishna, 2024). This issue has relevant practical implications, which can be categorized into two main areas. First, children need to be educated about digital communication, including its unique codes and, most importantly, the fundamental principles of this form of communication. Second, providing adolescents with physical spaces to engage in face-to-face interactions and socialize without smartphones is imperative. In contemporary society, smartphone usage is pervasive among adolescents, even in shared physical spaces (George and Odgers, 2015). Therefore, creating environments where smartphone use is minimized is essential. One potential setting for this could be schools, though this would require regulating mobile device use not only in classrooms but also in recreational areas such as playgrounds (Machmud, 2018). The data also support the relevance of extracurricular spaces—such as camps or artistic and athletic activities—where smartphones are restricted, allowing peer interaction to flourish (Lepp et al., 2015). These environments play a vital role in supporting the developmental process of children and adolescents, making it the responsibility of the educational and social community to provide them.

Another commonly cited benefit of smartphones is their role in facilitating contact between children and their parents in case of emergencies. Research indicates that many parents provide smartphones to their children primarily for this reason (Kildare and Middlemiss, 2017). While the need for parental contact—particularly as children transition toward independence—is understandable, the nature of these interactions should be carefully examined (Barron, 2014).

During early adolescence, children must develop the ability to navigate and resolve everyday challenges independently, especially when in public spaces. Parents play a critical role in fostering these skills by providing both autonomy and guidance. Therefore, children should not automatically rely on contacting their parents whenever they face difficulties. This principle should be clearly explained to parents through targeted training. If parent–child communication remains a priority, alternative tools to smartphones—such as mobile watches or non-smartphone devices without internet access—should also be considered. These devices use different applications with distinct risks (Mascheroni and Ólafsson, 2014). It is, therefore, essential to educate o educate families before children reach ages 9–11, about these alternatives and their respective benefits and risks to enable informed decision-making regarding the most appropriate device for their children.

Older children who own smartphones highlighted information-seeking as a key benefit. On the one hand, they frequently use their devices to search for information related to schoolwork. This raises the question of whether smartphones are the most suitable tool for educational purposes (Sung et al., 2016). Previous research, particularly during the pandemic, found that many children without access to computers were forced to rely on smartphones for learning, which hindered their educational experience (Wang et al., 2021). These findings align with the children’s responses in our study, which show that smartphone use for academic searches was closely associated with owning a personal device and being in higher grade levels.

Additionally, children with their own smartphones reported using them to search for information that they may feel uncomfortable asking others about or that is otherwise inaccessible to them. While the internet offers a wealth of valuable information, it also contains a considerable amount of false information. Children (and even adults) are often unable to distinguish between credible and unreliable sources (Metzger et al., 2015). Smartphones, therefore, become a gateway to information on sensitive topics, such as sexuality (Macharia et al., 2021). However, the lack of a regulatory framework for online information presents a major challenge, particularly in the context of sex education. This issue is exacerbated by the widespread availability of inappropriate content, such as pornography (Widman et al., 2021). Similar concerns apply to other topics, including drug use and radicalized ideologies, where unreliable or harmful sources are easily accessible (Holt et al., 2017). These risks reinforce the importance of integrating media and information literacy into early digital education.

In contrast, younger children who do not own smartphones emphasize two key benefits: alleviating boredom and providing multimedia entertainment. They highlight the role of smartphones in reducing feelings of restlessness, which can be linked to parents’ tendency to offer smartphones as a quick solution to keep children occupied and minimize disruptions (Yang et al., 2020). It is now common to see children using smartphones in public spaces such as restaurants, bars, and public transportation—even while seated in strollers (Levine et al., 2019). However, constantly filling moments of boredom with smartphone use may have unintended consequences. While prolonged boredom can negatively impact children’s mental health and well-being (Panova and Lleras, 2016), occasional boredom is beneficial. It fosters creativity (Mann and Cadman, 2014), encourages self-reflection (Belton and Priyadharshini, 2007), enhances attention and concentration (Malkovsky et al., 2012), and builds emotional resilience (Ghobadi et al., 2021; Isacescu et al., 2017). It also allows space for spontaneity and intuition—essential aspects of child development (Belton and Priyadharshini, 2007). Therefore, the effects can be significant if children are deprived of these opportunities and are constantly exposed to highly stimulating multimedia content. Indeed, some studies suggest that smartphone use negatively impacts creativity (Li et al., 2023; Olson et al., 2022). The data also support the relevance of extracurricular spaces—such as camps or artistic and athletic activities—where smartphones are restricted, allowing peer interaction to flourish. Such alternatives include family games (Wang et al., 2018) and direct contact with nature (Chawla, 2015).

In conclusion, regarding children’s training in smartphone use, it is important to note that formal instruction typically begins around ages 9–10, despite most children using smartphones—albeit those belonging to their parents—at a much younger age. Many scholars emphasize the importance of early digital literacy education (Bjørgen and Erstad, 2015; Erstad and Gillen, 2019; Neumann et al., 2017). In our study, only 38% of children reported having received digital training on multiple occasions, with younger children particularly underrepresented in this area. Providing digital education only at age 9 or later excludes younger users who are already engaging with devices, often without adequate guidance. Therefore, the timing and content of this training must be carefully designed, and this study offers several key areas—such as safe communication, information evaluation, and responsible use—that should be considered in digital education programs.

While this study has outlined practical implications, it also has certain limitations. The sample was drawn from a specific area in northern Spain (the Basque Country), which may limit the generalizability of the findings to other cultural or geographical contexts. Furthermore, only 26% of participating children owned a smartphone, which may introduce a bias in the results toward the experiences and perceptions of children who primarily use their parents’ devices. As such, future research should consider including older adolescents or secondary school students, who are more likely to have personal devices and a broader range of usage experiences.

Additionally, although we performed chi-square analyses to explore associations between categorical variables, the main analyses were conducted using IRaMuTeQ, a lexicometric tool based on Reinert’s method of descending hierarchical classification. This software does not produce conventional statistical indicators such as effect sizes or confidence intervals, as its focus lies in analyzing co-occurrences and semantic structures within textual data. This methodological characteristic imposes certain restrictions on the statistical interpretability of our results. Future research could complement this approach with additional statistical tools to enhance analytical depth.

Although smartphones offer undeniable benefits and appeal to children, it is essential to recognize that they are in a critical stage of development where their well-being must be carefully considered. Consequently, comprehensive digital literacy education is crucial for both children and their families. However, for this digital education to be truly effective, it must begin from the perspective of the children themselves — a key insight that has emerged from the findings of the present study.

## Data Availability

The raw data supporting the conclusions of this article will be made available by the authors, without undue reservation.
